# The Difficulty of Effectively Using Allocentric Prior Information in a Spatial Recall Task

**DOI:** 10.1038/s41598-020-62775-5

**Published:** 2020-04-24

**Authors:** James Negen, Laura-Ashleigh Bird, Eleanor King, Marko Nardini

**Affiliations:** Department of Psychology, Durham University, Durham, United Kingdom

**Keywords:** Cognitive neuroscience, Human behaviour

## Abstract

Prior information represents the long-term statistical structure of an environment. For example, colds develop more often than throat cancer, making the former a more likely diagnosis for a sore throat. There is ample evidence for effective use of prior information during a variety of perceptual tasks, including the ability to recall locations using an egocentric (self-based) frame. However, it is not yet known if people can use prior information effectively when using an allocentric (world-based) frame. Forty-eight adults were shown sixty sets of three target locations in a sparse virtual environment with three beacons. The targets were drawn from one of four prior distributions. They were then asked to point to the targets after a delay and a change in perspective. While searches were biased towards the beacons, we did not find any evidence that participants successfully exploited the prior distributions of targets. These results suggest that allocentric reasoning does not conform to normative Bayesian models: we saw no evidence for use of priors in our cognitively-complex (allocentric) task, unlike in previous, simpler (egocentric) recall tasks. It is possible that this reflects the high biological cost of processing precise allocentric information.

## Introduction

For readers who may be unfamiliar, we will start by defining *prior information, allocentric*, and *allocentric prior information*. *Prior information* is a description of the long-term statistical properties of a given domain or environment. For some examples: the price of a stock index tends to double about every 7.5 years; the average man in the United States is 5 feet and 9 inches tall; there tend to be more tornadoes in Kansas, but more earthquakes in California. When used by a decision-maker (e.g. to evaluate the probability of an earthquake), this long-term statistical information is referred to as *prior information*, or a *prior*. The *allocentric* frame of reference is used when a specific location is encoded by relating it to other stable locations in a given environment or region. For example, a globe is a way of organizing allocentric information; it can be used to find where any given country is located relative to any other country. Minimally, allocentric reasoning involves at least relating one location to one other stable landmark. For example, “My desk is near the door”. Allocentric information stands in contrast to egocentric information, which relates locations to the self. For example, “My desk is in front of me”. *Allocentric prior information* is any information that qualifies both as prior information and allocentric information. For example, “I tend to park my car near the security hut.” This is a description of a long-term statistical structure that relates one location (where I parked my car) to a stable external reference (the security hut). The central empirical aim of this study is to test whether people rapidly learn to use prior information during an allocentric spatial recall task. That is: whether they use allocentric prior information.

Questions about the use of priors are important because they are central to current theory about the fundamental principles that organize many parts of cognition. Recent work suggests that many aspects of human perception, memory, and decision-making are approximated well by Bayesian principles^1,2^. In other words, evolutionary pressures have led the human brain to process information in a way that is near-optimal for reducing uncertainty, following or approximating a Bayesian statistician. Part of this is the use of prior information about the environment. Many studies have found that people rapidly begin to exploit prior information in simple perceptual-motor tasks – for example, when judging the trajectories or speeds of items on a screen^3–5^ or the locations of hidden objects^6–14^. Over a longer timeline, this may also explain prototype effects in spatial recall^15^. In related studies, people use computations with additional hallmarks of Bayes-like reasoning^1,2,16–18^, including optimal cue integration^19–22^, causal inference^23,24^, and the ability to work correctly with an uneven utility function^25–27^ (i.e. a situation where some errors are more important to avoid than others). The Bayesian framework can also help explain effects seen in memory tasks as well. For example, when people try to remember recently-seen colours, their percepts are biased in a manner explained well by Bayesian combination of different strategies for remembering the exact colour^28^. Unifying a wide range of different findings under a single coherent theory has been a major step forward for our understanding of how the human mind functions.

To discuss this more clearly, it is helpful to create a *normative Bayesian observer*. This is a theoretical construct, the ideal of an organism that uses Bayesian principles perfectly to improve accuracy and precision. A normative Bayesian observer still has noise in different streams of perception, but the observer integrates the multiple sources of information available in the most efficient way possible. This has been applied, for example, to modelling how people use audio and visual information together during multisensory tasks^29^. Neither the visual nor the auditory system can perfectly locate where things are, so a normative Bayesian observer cannot either. However, a normative Bayesian observer can combine the two together in a very efficient way, benefitting strongly from the availability of both signals. Crucially for our purposes here, a normative Bayesian observer also exploits available prior information. For this paper, we treat this as part of the definition: a normative Bayesian observer, when placed in a situation with strong prior information, exploits that prior information in line with Bayesian principles. A full mathematical treatment is given here^16^. To phrase the paragraph above in these terms: there are many reports showing strong similarities between actual human participants and theoretical normative Bayesian observers^1–27^.

While there are many ways that human behaviour is similar to a normative Bayesian observer, there are also many reports of tasks and domains where the two strongly differ^30^. A key current problem is therefore to delineate and organize which parts of perception and cognition are similar to a normative Bayesian observer and which parts are not – and in what ways^31^. Working towards this is an important step towards elaborating and evaluating unified accounts of perception and cognition. It could also be a guide to studying the neural mechanisms that implement different perceptual and cognitive computations. For example, one recent theory suggests that the brain processes low-level sensory information like a normative Bayesian observer, without any similar mechanism in place for high-level abstractions^32–34^. The development of this type of theory benefits greatly from a wide catalogue of information about Bayesian reasoning across different, important kinds of cognition. Results are not yet available for allocentric spatial recall, which is an important everyday strategy for most adults. Further, allocentric information may be unusually biologically costly to compute, store, and work with; allocentric computations depend on the interplay of a large number of different, complex, specialized neural networks in the hippocampus and surrounding regions^35–39^, and allocentric recall is notoriously challenging during child development^40–43^ and for some adults^44–46^. Allocentric information may be an example where the cost of implementing Bayesian reasoning is too high compared to the gain. This makes it an interesting point of contrast with relatively low-level percepts like the motion of simple geometric shapes across a two-dimensional screen^3–5^. Allocentric prior information is thus an excellent case study to help better understand when human cognition operates like a normative Bayesian observer.

An important second reason to study allocentric prior use is to gain a better understanding of the specific problem domain of human spatial cognition in itself. The two major modes of spatial reasoning are egocentric (self-based) and allocentric (world-based). The two systems interact and combine in a variety of ways to adapt to the individual’s goals and the affordances of the local environment^47^. Of the two, allocentric information is more difficult and noisy but more durable and flexible^48–51^; purely egocentric information immediately requires updating or discarding whenever the viewpoint changes, but allocentric information can be used flexibly from any viewpoint. There have already been multiple studies suggesting that prior information in an egocentric frame can be used effectively after some exposure to a novel environment^6–14^. It is not yet known if the same is true for allocentric information. If so, this would indicate a capacity of human spatial cognition that has not yet been documented. If not, then there is an undocumented but fundamental difference in how egocentric versus allocentric information is processed. In particular, this would indicate that egocentric information is processed further than allocentric; it would mean that egocentric relations are analysed for useful prior distributions, but that allocentric relations are merely encoded and stored. Studying the use of allocentric prior information will result in a more complete characterization of human spatial cognition.

## Closely Related Studies and Results

To our knowledge, no previous projects have been designed to test if people can use an allocentric prior. However, there have been several reports of successfully using egocentric spatial priors^6–14^. In this kind of task, the participant is in front of a screen or other display device and has to point to a target. On each trial, they are presented with a noisy cue to the target’s location that is only relevant for that specific trial. In decision theory, and in these studies, such current information is generally referred to as a *likelihood*. In addition, targets tend to be in a specific area relative to the participant more often. That is the prior distribution. A normative Bayesian observer would appropriately balance the likelihood and the prior, maximising the chances of success^16^. In these studies, behaviour has often been in line with that expected for a normative Bayesian observer. Most crucially, participants exploit the prior information when responding. This can be measured as a bias in responses towards the centre of the prior distribution. The priors used in these kinds of tasks are usually thought of as sensorimotor priors – egocentric information about the way to move one’s own body without allocentric content.

In addition, there have been a few projects on the closely-related idea of allocentric probabilistic cueing^52–55^. For this kind of task, the participant is asked to search for a target and report something about it. There is a certain portion of the space where the target tends to be most often. This creates an opportunity for the participant to optimize their strategy: they can search in that portion of the space first and thus make their report faster on average. This is related to prior use in the sense that it involves tracking and making use of long-term statistics about the task’s environment (although there is no likelihood to use or balance). The typical result is almost paradoxical. Despite fluency with allocentric reasoning^47,51,56^ and being able to exploit probabilistic patterns from shortly after birth^57^, adult participants usually do not exploit an allocentric probabilistic cue^52–55^. In practice, this means that they do not adopt a strategy of searching the high-probability space first and thus do not make their reports faster. Based on this, we might expect that adults would not use allocentric priors – it seems like it could only be more difficult, since it involves also balancing a likelihood function.

However, there are three exceptions to the general trend above. First, one experiment did find some evidence of using an allocentric probabilistic cue^55^, specifically when a portion of the space was also cued with a different colour. Second, another found positive results in a large-scale environment when participants started from the same place but in random directions on each trial^58^. It is arguable whether this represents the use of an allocentric frame, since it still allows the same view of the space after turning. However, it does require something more than the most basic kind of egocentric reasoning. Third, another study found positive results when the cue was object-centred (e.g. usually in the top part of the object)^59^, which could arguably be thought of as a type of local or intrinsic allocentric frame^60^. Each of these suggests that allocentric probabilistic cue use can be achieved under the right circumstances.

In short, we have no direct evidence to see if rapid allocentric prior use occurs or not, and mixed reasons to expect that it may or may not from closely related studies.

## The Present Study

**Figure 1:**
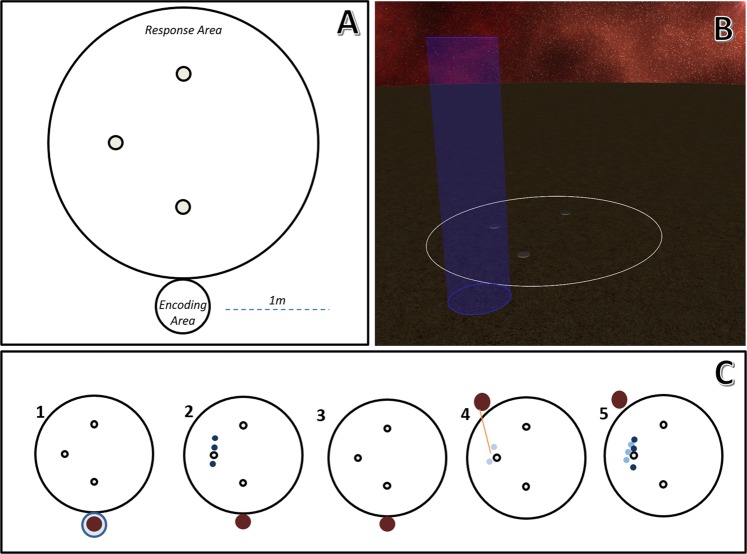
The virtual environment and procedure. (**A**) An overhead diagram of the environment. The small circles are stable landmarks. (**B**) A perspective screenshot of the virtual simulation. The large blue column is the encoding area. (**C**) The five steps to the procedure. Participants (brown marker) stood in the blue column, which was the encoding area (1). Three targets appeared (dark blue; 2). The targets disappeared (3). The participant’s viewpoint was changed and they were asked to point where the three targets had been (4). The participant received feedback on their responses (light blue) by seeing the actual targets again (5).

To fill this gap in the literature, we created a new task to test for allocentric prior use. Participants saw three targets (small spaceships) appear in a sparse environment in immersive virtual reality (Fig. 1). These disappeared. The participant’s viewpoint changed, forcing them to engage allocentric reasoning^40,51,61^. They then had to point where the three targets were in the environment. The prior distribution had a relatively low variance, limiting potential targets to an area <75 cm in radius. The environment also contained three small, identical, symmetric beacons arranged in an isosceles triangle. This arrangement is relatively difficult to use (no single beacon is enough to remember exact locations), which was done to induce a relatively high variance in their memory (the likelihood). A relatively high variance in the likelihood and a relatively low variance in the prior distribution is the situation where exploiting the prior distribution can have the largest impact^13^. Participants saw and tried to recall 90 targets in each of two halves of the testing session. We considered the first half as an initial opportunity to adapt to the presence of the prior. We analysed performance in the second half for evidence of using this distribution. Our central question is whether adult participants will show a reliable allocentric prior use effect. Answering this will further illuminate when we can use normative Bayesian observers to understand and predict human behaviour. It will also clarify exactly how we use allocentric information.

We had three hypotheses:

### Rapid allocentric prior use

As the experiment progresses, participants learn to use the available prior distribution. On each trial, their memory of the locations will inevitably have some noise. Participants begin to use the prior by biasing their responses towards locations that have been more likely to contain a target on previous trials. This results in their responses, on average, being closer to the prior mode (the place where targets are most likely) than the targets themselves. We expect this specifically in the second half of the testing session (after there has been sufficient opportunity to adapt to the prior). The mathematical underpinnings of this are generally well-known in the literature^16^, but just to be sure that this result is expected within the parameters of our experiment, we also wrote a short simulation script in Matlab (Supplementary Note – Prior Use Simulation). The results confirm that a normative Bayesian observer would reduce error by biasing their responses towards the prior mode. (The exact extent of this bias depends on the noise in memory – more noise leads to more bias.) If this is also seen in humans, that result would confirm that humans can use allocentric prior information like a normative Bayesian observer.

### Non-Learned allocentric biases

Participants do not learn to use the prior, but do come into the experiment with certain biases anyway. In particular, the local beacons make for an obvious reference point to use for the representation of a target location. Participants might be conservative about responding too far away from the base reference point that they use. This results in biases towards the local beacons, but not due to any prior use. The responses should be closer, on average, to the beacons than the targets. Separating this hypothesis from the one above is the reason why we tested four different priors in different conditions, but with the same configuration of beacons: so that we can see which is consistently biasing responses across conditions. This result would allow us to examine the allocentric biases that occur regardless of the prior. It would also represent a limiting case where a normative Bayesian observer is not a particularly useful way to understand and predict behaviour.

### Null hypothesis

Participants do not enter the experiment with any biases and do not learn any. There is noise in where participants place their responses, but nothing systematically biasing them towards the prior modes nor the beacons. This would also represent a case where human behaviour is not appropriately modelled as a normative Bayesian observer.

## Method

### Participants

Forty-eight adult participants were recruited from Durham University (12 per condition). Their ages ranged from 18 to 29 years old. Participants were given either £8 or an hour towards a credit system where students volunteer in each other’s studies. Participants were also required to either have normal vision or be able to correct their vision to normal with contact lenses. Informed consent was obtained from all participants in written form. The study was approved by Durham University Psychology’s Ethics Committee (approval reference PGT2018MN2). The research was performed in accordance with the relevant guidelines and regulations.

### Apparatus

Participants wore an Oculus Rift headset (Consumer Version; Menlo Park, CA, USA). The headset was tracked continuously by small reflective markers feeding into a Vicon Bonita motion tracking system (Oxford, UK). They were also given a motion-tracked ‘wand’ for indicating responses, made of some PVC and a screwdriver handle. The virtual environment was programmed in WorldViz Vizard 5. With real-time updating of the participant’s position and headset’s position from the motion capture markers, participants were able to freely traverse a space of about 5 m × 10 m, with approximately 3 m × 3 m used during this study.

The virtual environment was very sparse (see Fig. 1A,B). There was a floor with a sand texture. There was a skybox, rendered at infinite distance, with the appearance of an outer space nebula. A circle was placed with a radius of 1.3 m. Using the center of the circle as (0, 0), three small beacons were placed at (−0.65, 0), (0, 0.65), and (0, –0.65) with distances in meters. These beacons were grey cylinders with a height of 1 cm and a radius of 6.5 cm. A translucent blue column was placed to indicate where the participant should stand. When standing inside this column, it became invisible. The wand was rendered as a rectangular solid with a sphere on the end and a thin column of light extending along its main axis (like a laser pointer).

### Stimuli

**Figure 2:**
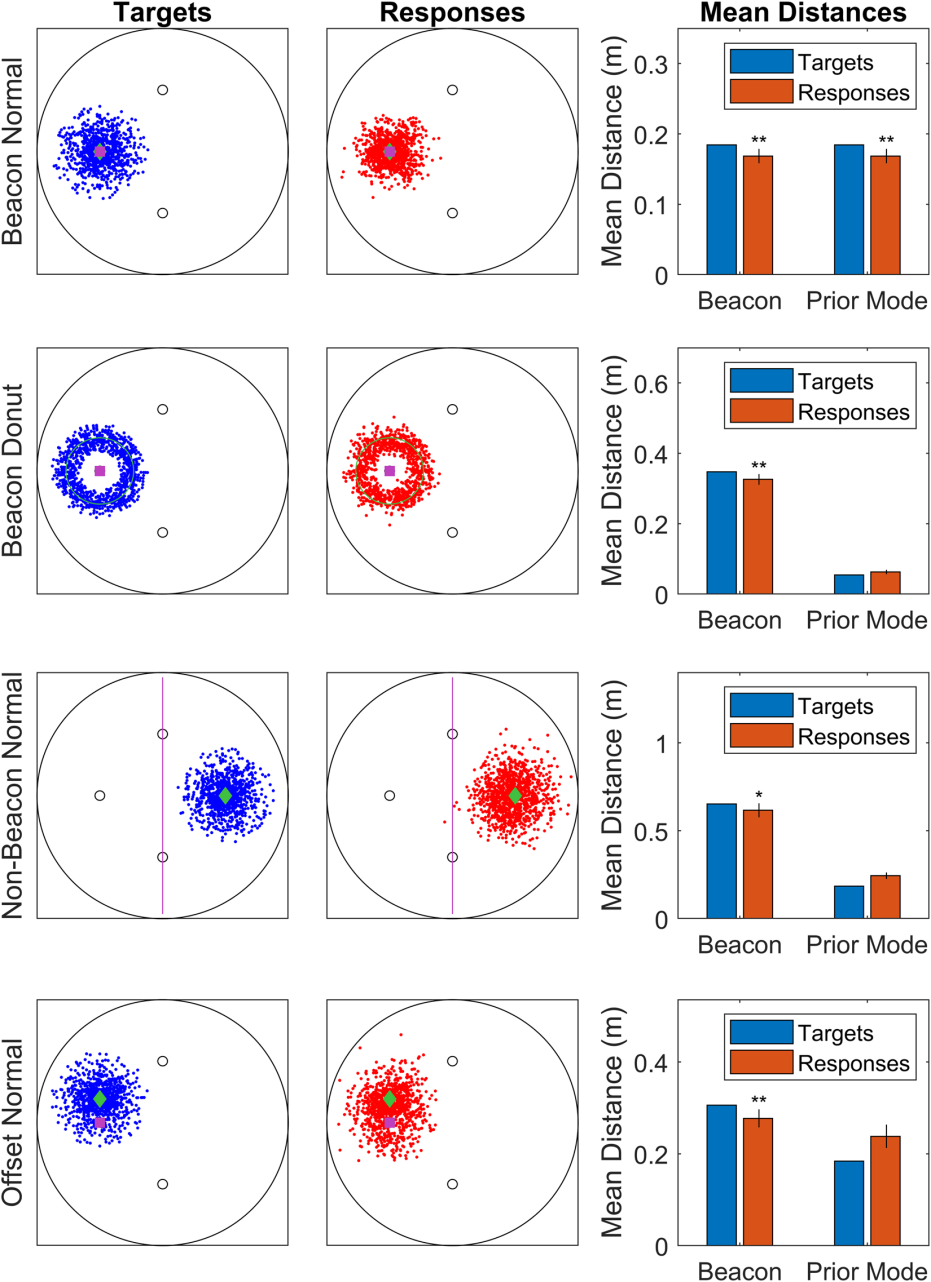
The distribution of targets, responses, and the biases shown. The left column shows the distribution of targets. The targets themselves are the blue dots (opacity 25%). The purple item (square or line) shows where we would expect the bias to point if it were controlled by the local beacons. The green item (diamond or circle) is placed on the prior mode, the place (or set of places) with the highest prior probability. The middle column shows the distribution of responses. On the right, distances to the beacon (left) or prior mode (right) can be seen for both targets (blue) and responses (orange). In essence, a shorter orange bar means a bias towards that feature, and a bias towards the prior mode would suggest allocentric prior use was occurring. Error bars are 95% confidence intervals. *p < 0.05; **p < 0.01.

The stimuli were three small “spaceships”, dark blue disks with a radius of 3 cm and some small white lights along their rim. Three targets were presented on each trial to increase the likelihood variance. Their positions were drawn from a prior distribution depending on the condition (also see Fig. 2, left column):

- **Beacon Normal:** targets were drawn from a bivariate normal distribution with a mean on top of the beacon at (−0.65 m, 0 m), the one to the left in figures here. It had a covariance of zero, and a standard deviation of 15 cm along both axes. This is a baseline to see if we observe any biases at all, as both the local beacon and the prior mode are in the same distance and direction. Biases towards the local beacon would be consistent with either Rapid Allocentric Prior Use or Non-Learned Allocentric Biases.
- **Non-Beacon Normal:** targets were drawn from a bivariate normal distribution with a mean at (0.65 m, 0 m), again a covariance of zero, and the same standard deviation of 15 cm. This places the mean at the place where the fourth beacon would be to form a square, to the right in figures. This a prior with a kind of implied marking but no actual beacon. Non-learned Allocentric Biases predicts that the responses will be biased in a negative direction along the x-axis (i.e. left in figures, towards the beacon array). Rapid Allocentric Prior Use predicts a bias towards the prior mode at (0.65 m, 0 m).
- **Beacon Donut:** targets were drawn in polar coordinates centred on the left beacon. The angle was uniformly distributed. The distance from the center of the beacon was an average of 35 cm and a standard deviation of 7 cm. This creates a kind of ‘donut’ shape. This a prior that can be related easily to a local beacon, but is not directly on it. Non-Learned Allocentric Biases predicts a bias towards the beacon at (−0.65 m, 0 m). Rapid Allocentric Prior Use predicts a bias towards the prior mode, which is a circle with a radius of 35 cm and a center at (−0.65 m, 0 m).
- **Offset:** targets were drawn from a bivariate normal distribution with a mean at (−0.65 m, 0.25 m). In other words, it was offset from a beacon so that it fell slightly above the left beacon in figures. It again had a covariance of zero and a standard deviation of 15 cm. This is a direct conflict between bias towards the prior mode and bias towards the beacon, as many targets fall in between and can only move towards one or the other. Non-Learned Allocentric Biases predicts a bias towards the beacon at (−0.65 m, 0 m). Rapid Allocentric Prior Use predicts a bias towards the prior mode at (−0.65 m, 0.25 m).

For the first 30 trials (totalling 90 targets), the exact target locations were random but constrained to fall within the inner 98% of the distribution. The remaining 30 trials, which are the main subject of the analysis (after they have had some experience to learn to use the prior), used the same 90 locations as the first 30 trials. However, for the last half, the locations were randomly re-grouped into sets of three and then randomly re-ordered. This was done to ensure that the prior participants had the opportunity to learn during the first half was exactly correct for the second half.

### Procedure

The goal of the game was explained to the participant. The participant was then fitted with the headset. On each trial, five things occurred (Fig. 1C). First, they stood within the blue column. Second, the three targets appeared simultaneously. These targets were drawn from a distribution as described in *Stimuli*. Third, the targets disappeared after being visible for one second. Fourth, there was a brief disorientation to disrupt egocentric relations. The screen faded to black over 1.25 seconds, the participant’s viewpoint inside the simulation was changed, and the screen faded up from black over another 1.25 seconds. The experience of this is like being ‘teleported’, creating a need to recover a sense of heading and direction. The displacement amounts were in even steps from 1/8^th^ to 7/8^th^ of the way around the circle, in random order. They used the wand to point to three locations where they judged the targets had been. Participants entered a response by clicking a small handheld mouse. Fifth, the actual targets would re-appear while their responses were still visible. When the mouse was clicked again, the previous targets and responses would disappear for one second before the next trial’s targets appeared. (In other words, the next trial started where the last one ended). In total, there were 60 trials.

### Data analysis plan

Responses from the first 30 trials (90 exposures) were not analysed. Those were considered to be the time when participants could learn to use the prior. Only the last 30 trials (90 exposures) were analysed.

Within each condition, for each participant, four summary statistics were calculated:

1. Average distance between the prior mode and the targets. For all conditions except *Beacon Donut*, the prior mode was the center of the bivariate normal prior distribution. For the Beacon Normal, the prior mode was at (−0.65 m, 0 m); for Non-Beacon Normal, it was at (0.65 m, 0 m); for Offset, it was at (−0.65 m, 0.25 m). For these conditions, the average distance between these points and every target was calculated. For *Beacon Donut*, the mode of the prior distribution is a circle with a radius of 35 cm and a center at (−0.65 m, 0 m). For this condition, the distance between the prior mode and the targets was calculated by first finding the distance between the center of the circle and the target, then taking the absolute difference between that figure and 35 cm. This gives the distance to the nearest point on the circular prior mode.
2. Average distance between the prior mode and the responses. This was calculated the same way as #1, except using responses instead of targets. This statistic, subtracted from #1, is the overall bias towards the prior mode. A significant positive bias here would support the Rapid Allocentric Use hypothesis.
3. Average distance between the local beacons and the targets. For all conditions except *Non-Beacon Normal*, this is the average distance between the beacon at (−0.65 m, 0 m) and the targets. For *Non-Beacon Normal*, the array of three beacons are all leftwards in figures (negative along the x-axis). The average distance to the beacons is the average x-axis difference between each target and x = 0.
4. Average distance between the local beacons and the responses. This was calculated the same way as #3, except using responses instead of targets. This statistic, subtracted from #3, is the overall bias towards the local beacons. A significant positive bias here would support the Non-Learned Allocentric Biases Hypothesis.

## Results

Every condition resulted in a small but statistically significant bias towards the local beacons. We only observed a bias towards the prior mode when the prior mode was directly on top of a local beacon (‘Beacon Normal’ condition). This favours Non-Learned Allocentric Biases; it suggests that this kind of allocentric spatial memory is subject to reliable biases, but these biases are not the result of rapidly learning to use an allocentric prior.

The median distance between the centroid of the targets and the centroid of the responses was 11.71 cm (25^th^ percentile at 6.51 cm, 75^th^ at 18.50 cm). A total of 41 trials (2.85%) were excluded as outliers. These trials had targets that were nearest the beacon at (−0.65 m, 0 m), the one on the left in figures, but a response that was nearer one of the other two beacons.

For the Beacon Normal condition, responses were biased towards both the local beacon and the prior mode in a one-tailed t-test, t(11) = 3.47, p = 0.003, mean difference of 0.016 m, Cohen’s d = 1.001. As Fig. 2 shows, the response distribution is compressed in towards the left beacon, compared to the target distribution. This is consistent with either Non-Learned Allocentric Biases or Rapid Allocentric Prior Use, but not the Null Hypothesis.

For the Beacon Donut condition, responses were biased towards the nearest beacon, t(11) = 3.14, p = 0.005, mean difference of 0.022 m, Cohen’s d = 0.907. However, the responses were not biased towards the prior mode, t(11) = −3.12, p = 0.995, mean difference of −0.009 m, Cohen’s d = −0.901. As Fig. 2 shows, the response distribution is compressed in towards the left beacon, away from the prior mode, compared to the target distribution. This is supports the Non-Learned Allocentric Biases hypothesis, does not support the Rapid Allocentric Prior Use hypothesis, and violates the Null Hypothesis.

In the Non-Beacon Normal condition, the idea of moving towards the beacon is captured by just moving leftwards (towards the purple line) in Fig. 2. The responses were biased towards the beacons, t(11) = 1.98, p = 0.037, mean difference of 0.036 m, Cohen’s d = 0.571. However, responses were not biased towards the prior mode, t(11) = −7.05, p > 0.999, mean difference of −0.059 m, Cohen’s d = −2.034. As Fig. 2 shows, the response distribution is pushed leftwards, away from the prior mode but towards the beacon array, compared to the target distribution. This is supports the Non-Learned Allocentric Biases hypothesis, does not support the Rapid Allocentric Prior Use hypothesis, and violates the Null Hypothesis.

In the Offset condition, responses were biased towards the beacon at (−0.65 m, 0 m), the one on the left in figures, t(11) = 3.25, p = 0.004, mean difference of 0.029 m, Cohen’s d = 0.937. Responses were not biased towards the prior mode, t(11) = −4.67, p > 0.999, mean difference of −0.054 m, Cohen’s d = −1.349. As Fig. 2 shows, the distribution of responses is, on average, pushed downwards towards the beacon and away from the prior mode. This is supports the Non-Learned Allocentric Biases hypothesis, does not support the Rapid Allocentric Prior Use hypothesis, and violates the Null Hypothesis.

In designing the study, we wanted to avoid situations where optimal prior use would have very little effect and/or the analysis is likely to miss a more substantial effect. Towards the former goal, we wanted to avoid a situation where the prior has a great deal more spread than the likelihood. We calculated that the average distance from the prior mode to each target was 5 cm in the Beacon Donut condition and 19 cm in all others. We also calculated the average distance from targets to responses, using the pairing that minimized error (each trial had three targets and three responses, so there are six possible pairings). This was 21 cm for Beacon Donut, 20 cm for Non-Beacon Normal, and 20 cm for Offset Normal. This suggests that the experimental design did indeed have meaningful scope for participants to make use of the prior in the three conditions that did not show a bias towards the prior mode. Further, confidence intervals for bias toward the prior mode were 1–5 cm wide and lay entirely below zero for these three conditions (Fig. 2). This suggests that the null results (in terms of a positive bias towards the prior mode) are unlikely to be due to issues with statistical power or imprecise estimation.

## Discussion

The biases we observed were consistently pointed towards the local beacons. The biases were only toward the prior mode when the prior mode was directly on a beacon. If responses were subject to a Bayesian process, we would have expected to see a consistent bias towards the prior mode. In the conditions where the prior mode was not directly on a beacon, confidence intervals actually lay entirely on the opposite size of zero (i.e. biases away from the prior mode) for such an effect. This strongly favours the Non-Learned Allocentric Biases hypothesis: the way participants use allocentric information is not consistent with the predictions of a normative Bayesian observer, at least in the present task, but is still subject to reliable biases.

We should note carefully that this does not mean that adults do not use spatial priors. Egocentric prior use has been demonstrated several times in adults^6–14^. Earlier, we used an example of learning that your car tends to be parked near the security hut. While that allocentric prior may be difficult to exploit, it might still be very easy to learn to exploit a similar egocentric relation. For example, maybe your car tends to be to your left when you come out of the main exit. If you come out of the main exit every day, that could be just as useful. The results also do not mean that participants were unaware of the prior. Being aware that the targets tend to group in one place is different from using that pattern to create a small bias that can reduce error.

From our point of view, one of the more interesting facets of egocentric prior use is the way that participants can accomplish it without ever being told what the prior distribution is – or even being told that any prior distribution exists. Instead, they learn to use the prior from exposure to samples from it^6–14^. The current experiment was run in a similar manner; we made design decisions to make the prior potentially very useful and clear, but chose not to inform participants of its existence. We also chose not to show the full prior distribution, but instead just showed samples from it. To us, this better reflects the way that perception was used in the evolutionary environment and also reflects our research focus. However, it is possible that a similar experiment where participants are informed of the prior distribution may find that participants do exploit the prior. This would be a possible way to extend the current claim – a failure to use available allocentric priors – into a more specific investigation of whether certain task parameters can support a different outcome.

One persistent issue with measuring prior use is the question of how long the experimental session should continue. In other words, if a negative result is obtained, one can always object that prior use may have occurred if the participant had more time to learn how to use it. At some point, we have to decide what kinds of timeframes we are interested in studying. As experimenters choose to study what happens with more and more exposure to the prior, they are focusing on phenomena that have less and less opportunity to be expressed in everyday life. As a comparison, egocentric prior use has been shown to adapt to new priors that are shown in blocks of only 100 exposures^12^, often within the first half of the block. Further, allocentric probabilistic cue use has been shown in only 80 exposures^55^. Here we chose to extend that to a total of 180 exposures. This should have been ample time, at least if the mechanisms involved are similar. Further, the trials were arranged so that an ideal learner would have an exactly correct point estimate of the prior distribution’s mean and variance at the midpoint of the study.

The present results illuminate a situation where a normative Bayesian observer is not a particularly useful way to understand behaviour, predicting specific patterns that are not reflected in observed behaviour. On one hand, it is clear that prior information is a very useful construct when understanding things like simple reaching behaviours^13^ and many other psychological phenomena^1,2,16^. On the other hand, we show here that it has limited use in understanding how people recall locations in an allocentric frame. This provides a counterpoint to a current debate over the nature of Bayesian models. Such models have been called “just-so stories”^62^, argued to have virtually no predictive power and too much flexibility to fit any provided data. It seems extremely difficult to imagine how the present data could ever be construed as showing an effect of prior use. Many studies are starting to document not just the ways that behaviour is similar to a normative Bayesian observer, but the ways that it is not^30^, which is just as important. This also highlights a larger question: what is the common link between this task and other tasks where behaviour appears non-Bayesian? Ultimately, more research will be needed to find out. For now, the present data are consistent with an emphasis on Bayesian models for processing low-level sensory data^32–34^ rather than higher-level abstractions like allocentric information.

These results also emphasize the biological cost of sophisticated allocentric reasoning and the evolutionary compromises that result from it. People often find allocentric reasoning difficult, especially as children^40–43^ but even as adults^44–46^. Due to recent advances in Neuroscience, the kind of cost associated with a higher-resolution representation is relatively well understood. The process of creating an allocentric representation relies on multiple different networks in the mammalian brain^35–37^. One key element of this is the grid cells, a kind of cell in the hippocampus and surrounding areas that fire in a hexagonal grid throughout a space^38,39^. These cells engage either when moving to a place or just imagining it^63^. Networks of grid cells operate at different spatial frequencies^35^. Having a more refined memory of different places throughout a space requires more networks of these cells to cover finer distinctions. At some point, the biological investment required to track a small spatial distinction is going to be greater than the return the organism acquires^64,65^. What we observed here reinforces the view that allocentric reasoning is “good enough” but not optimal in two different ways.

First, and most obvious, we did not observe allocentric prior use. The kinds of computations needed to exploit priors are not well understood, but they virtually must add complexity and biological cost. The process of evolution appears to have favoured the simpler strategy of using egocentric priors, at least for relatively short time frames.

Second, we observed reliable but small allocentric biases. While all four conditions showed a significant bias towards the local beacons, considering the size of the effect becomes important here. It was only about 1–3 cm. For a species that is about 160 or 170 cm in height on average, it is not obvious when a bias of that size would matter.

In the future, it might be possible to do a close variant of the current study but with egocentric prior use. That could serve as a way to directly compare egocentric and allocentric prior use. This might become somewhat complex. On one hand, it is not obvious how to allow egocentric priors while retaining the perspective switch. On the other hand, it is not obvious how to make a direct comparison if the perspective switch is removed, since it is one of the main ways that memory noise is induced. In addition, to some researchers, it might be more important to focus on where and how the process of learning and using an allocentric prior breaks down – for example, to see if there is any evidence that participants understand the prior distribution’s parameters but still fail to apply the prior to their responses. In addition, it would be good to make sure that similar results are obtained in a wider variety of environments.

To summarize, we found no evidence that adults will rapidly learn to use a spatial prior if they have to use the allocentric frame of reference to do so. There are biases present in their responses, but these appear to be biases towards local beacons rather than biases due to the exploitation of priors. We interpret this as a limiting case where normative Bayesian models are not particularly useful for understanding behaviour and also interpret this as a key difference in how egocentric versus allocentric information is processed.

## Supplementary information

Supplementary information.

Supplementary information 2
