# Supplementary material for: The Difficulty of Effectively Using Allocentric Prior Information in a Spatial Recall Task
Source: Sci Rep. 2020 Apr 24;10:7000. doi: 10.1038/s41598-020-62775-5 (PMC7181880; doi:10.1038/s41598-020-62775-5)
Supplement: Supplementary file 1 — Supplementary information. [file 41598_2020_62775_MOESM1_ESM.docx]

Supplementary Note – Prior Use Simulation

James Negen, Laura-Ashleigh Bird, Eleanor King, and Marko Nardini

This document accompanies *The Difficulty of Effectively Using Allocentric Prior Information in a Spatial Recall Task*.

The Rapid Allocentric Prior Learning hypothesis conceptually states that participants learn and apply the prior distribution of targets in an allocentric frame of reference. This is done to reduce the amount of error in the responses. In practice, this means that we expect a specific bias to appear on average. We expect the responses to be closer to the prior mode than the targets. The following supplement presents code and simulation results to confirm that an observer can reduce error by biasing responses towards the prior mode under conditions that are similar to the experiment.

The code was written in Matlab:

%This script runs a simulation of Bayesian reasoning in the case where a

%participant has learned the Beacon Offset prior and is using it optimally.

%This involves forming a memory with some noise around the target, then

%biasing their responses slightly away from their memory and towards the

%prior mode. As the simulation shows, this reduces error.

clear; clc; close all

nTrials = 100000;

PriorMean = [-.65,.25]; %as in Beacon Offset

PriorVariance = [.15^2, 0; 0, .15^2];

MemoryVariance = [.075^2, 0; 0, .075^2];

PriorPrecision = PriorVariance(1)^-1;

MemoryPrecision = MemoryVariance(1)^-1;

%In the case of normal distributions, Bayesian reasoning weights the prior

%and the likelihood (memory) by their precisions (1/variance). This leads

%to the highest (additive) precision around the targets.

PriorWeight = PriorPrecision / (PriorPrecision+MemoryPrecision);

Targets = mvnrnd(PriorMean,PriorVariance,nTrials); %as in Beacon Offset

Memories = mvnrnd(Targets,MemoryVariance);

Responses = repmat(PriorMean,nTrials,1) .* PriorWeight + ...

Memories .* (1-PriorWeight); %A weighted average of the prior mode (which is also the prior mean) and the memory

MemoryError = mean( (Targets(:,1)-Memories(:,1)).^2 +(Targets(:,2)-Memories(:,2)).^2 );

ResponseError = mean( (Targets(:,1)-Responses(:,1)).^2+(Targets(:,2)-Responses(:,2)).^2 );

subplot(1,2,1)

bar([MemoryError,ResponseError])

set(gca,'XTickLabel',{'Memory','Response'}); ylabel('Average Error (m)')

%By biasing responses towards the prior mode, error is reduced (left

%graph).

TargetDist = mean( (PriorMean(1)-Targets(:,1)).^2 +(PriorMean(2)-Targets(:,2)).^2 );

ResponseDist = mean( (PriorMean(1)-Responses(:,1)).^2 +(PriorMean(2)-Responses(:,2)).^2 );

subplot(1,2,2)

bar([TargetDist,ResponseDist])

set(gca,'XTickLabel',{'Target','Response'}); ylabel('Average Distance to Prior Mode (m)')

%This leads to the responses being closer than the targets to the prior

%mode on average (right graph).

In the simulation, the average error in memory is 0.0112m (Figure S1). Biasing the responses towards the prior mode reduces the error in responses to 0.009m (a 20% reduction).

Figure S1. A simulated optimal observer biases responses towards the prior mode (distance to responses is on average less than the distance to targets), resulting in less error in the responses than in the simulated memory.
